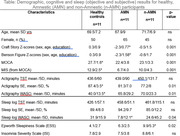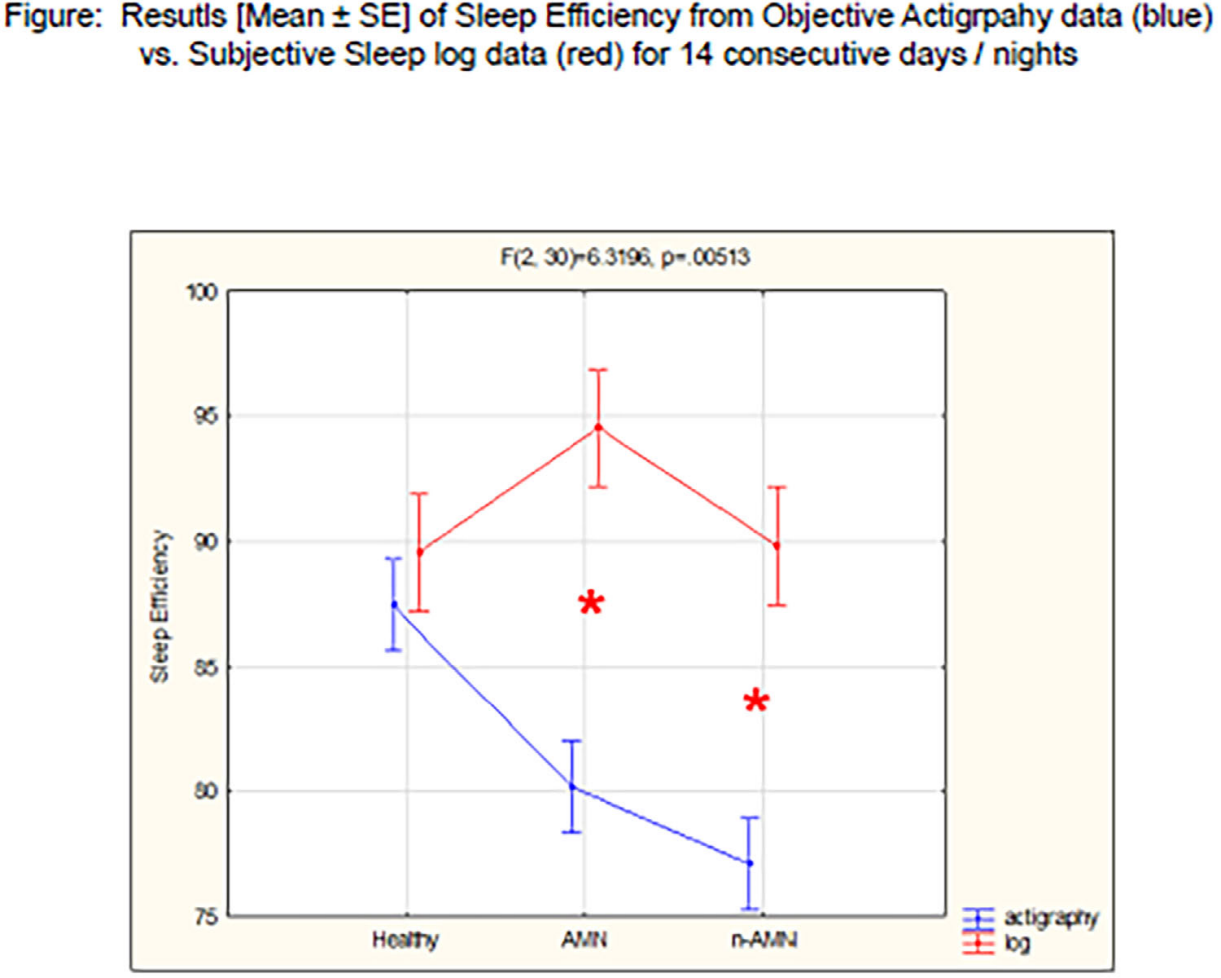# Objective and subjective sleep quality in Amnestic and Non‐Amnestic Elderly Patients: is a subjective report from the patient an accurate?

**DOI:** 10.1002/alz.092414

**Published:** 2025-01-03

**Authors:** Valentina Gumenyuk, Thomas Roth, Oleg Korzyukov, Nicholas Miller, Tristan Jones, Matthew Rizzo, Daniel L Murman

**Affiliations:** ^1^ 94480 Nebraska Medical Center, Omaha, NE USA; ^2^ Henry Ford Hospital, Detroit, MI USA; ^3^ UNMC, OMAHA, NE USA; ^4^ UNMC, Omaha, NE USA; ^5^ University of Nebraska Medical Center, Omaha, NE USA

## Abstract

**Background:**

Sleep disturbances and cognitive impairments, especially memory deficiency are one of the most common complications affecting everyday life in patient diagnosed with neurodegenerative disorder. Clinically evaluated, specific cognitive processes can be grouped into two categories: amnestic functions (AMN, clinically memory impairments) and non‐amnestic functions (n‐AMN, cognitive impairments non‐memory related). To date, no cure treatments are available for AMN / n‐AMN patients, therefore maintaining the well‐being and an adequate sleep quality of people who are in prodromal state (i.e., AMN or n‐AMN is a high priority for society general and for the patient / family specifical. In this study, we compared objective sleep results to self‐reported sleep in AMN and n‐AMN groups with respect to healthy matched controls to investigate whether the self‐reported sleep results are accurate

**Method:**

Sleep disturbances and cognitive impairments, especially memory deficiency are one of the most common complications affecting everyday life in patient diagnosed with neurodegenerative disorder. Clinically evaluated, specific cognitive processes can be grouped into two categories: amnestic functions (AMN, clinically memory impairments) and non‐amnestic functions (n‐AMN, cognitive impairments non‐memory related). To date, no cure treatments are available for AMN / n‐AMN patients, therefore maintaining the well‐being and an adequate sleep quality of people who are in prodromal state (i.e., AMN or n‐AMN is a high priority for society general and for the patient / family specifical. In this study, we compared objective sleep results to self‐reported sleep in AMN and n‐AMN groups with respect to healthy matched controls to investigate whether the self‐reported sleep results are accurate

**Result:**

Table summarizes all results found in the study. AMN group showed lower score in cognitive memory related processes (Craft story and Benson figure recall), as compared to n‐AMN and Healthy groups. Both patient groups overestimated their SE in self‐report data as compared to SE in objective actigraphy data. In healthy group, the SE was accurately self‐reported (see Figure).

**Conclusion:**

Our results suggest that regardless of the severity of memory impairments objective measure of sleep should be implemented for clinical evaluation.